# Being cool: how body temperature influences ageing and longevity

**DOI:** 10.1007/s10522-015-9571-2

**Published:** 2015-04-02

**Authors:** Gerald Keil, Elizabeth Cummings, João Pedro de Magalhães

**Affiliations:** Integrative Genomics of Ageing Group, Institute of Integrative Biology, University of Liverpool, Liverpool, L69 7ZB UK

**Keywords:** Metabolism, Neuroendocrine system, Life-extension, Rate of living theory, Thermodynamics

## Abstract

Temperature is a basic and essential property of any physical system, including living systems. Even modest variations in temperature can have profound effects on organisms, and it has long been thought that as metabolism increases at higher temperatures so should rates of ageing. Here, we review the literature on how temperature affects longevity, ageing and life history traits. From poikilotherms to homeotherms, there is a clear trend for lower temperature being associated with longer lifespans both in wild populations and in laboratory conditions. Many life-extending manipulations in rodents, such as caloric restriction, also decrease core body temperature. Nonetheless, an inverse relationship between temperature and lifespan can be obscured or reversed, especially when the range of body temperatures is small as in homeotherms. An example is observed in humans: women appear to have a slightly higher body temperature and yet live longer than men. The mechanisms involved in the relationship between temperature and longevity also appear to be less direct than once thought with neuroendocrine processes possibly mediating complex physiological responses to temperature changes. Lastly, we discuss species differences in longevity in mammals and how this relates to body temperature and argue that the low temperature of the long-lived naked mole-rat possibly contributes to its exceptional longevity.

## Introduction

The second law of thermodynamics states that within a closed thermodynamic system the entropy will increase over time until it reaches thermodynamic equilibrium. This increase in molecular entropy over time within living organisms has been proposed, under the assumption that the second law of thermodynamics applies to open systems, to increase susceptibility to age-related disorders and thus essentially equate, at a high level of abstraction, to the ageing process (Hayflick 2007). Other authors have argued that ageing is due to an increase in molecular disorder due to an increase in thermodynamic entropy (Demetrius 2013). One of the factors known to affect thermodynamics is temperature, and therefore it has been long speculated that it may influence the ageing process with organisms ageing faster at higher temperatures due to more molecular damage being generated (Conti 2008; Liu and Walford 1972; Rikke and Johnson 2004).

Temperature is an essential property of biological systems and various studies in many species have associated temperature with ageing and longevity. Early studies focused on animals that rely on external sources of heat (ectotherms) and whose internal temperature, because it depends on environmental conditions, can vary considerably (poikilothermy). A century ago, Loeb and Northrop showed that lifespan correlates negatively with temperature in fruit flies *Drosophila melanogaster* (Loeb and Northrop 1916). Another early study to observe the effects of temperature on longevity was in the cladoceran crustacean *Daphnia magna* (MacArthur and Baillie 1929). Later studies in other poikilotherms (in particular fishes), demonstrated that even mild changes in temperature over long periods of time can influence lifespan (Walford and Liu 1965). Important contributions to this field were made by Walford and Liu who pioneered the use of the South American fish *Cynolebias* as a model organism in laboratory temperature and longevity studies (reviewed in Rikke and Johnson 2004). This was then further explored in species that can generate their own body heat (endotherms) and usually maintain a relatively constant body temperature (*T*_*b*_), also known as homeotherms, like mice whereby marijuana derivatives were used to induce hypothermia. However, this approach was unable to reduce *T*_*b*_ long-term and thus was unable to properly establish the effect of temperature on mouse longevity (Rikke and Johnson 2004). Walford instead turned his attention to the body temperatures of great yoga masters in India which, despite the low sample size, showed that a low calorie intake could reduce *T*_*b*_ by 1–2 °C (Walford 1983). This low calorie intake was then principally used by him to induce low *T*_*b*_ in homeotherms to investigate the effects of core *T*_*b*_ on longevity (reviewed in Rikke and Johnson 2004).

Since these early pioneering studies into temperature and longevity, more recent studies have continued to expand these ideas by using various other species as models, with further consideration of the relationship between life-extending interventions such as caloric restriction (CR) and *T*_*b*_. Here, we review the literature on how temperature affects ageing and longevity from poikilotherms to homeotherms, including rodent models of life-extension. Possible mechanisms are discussed as well as the potential role of a low body temperature on the exceptional longevity of the naked mole-rat.

### Life-extension and temperature

**Fig. 1:**
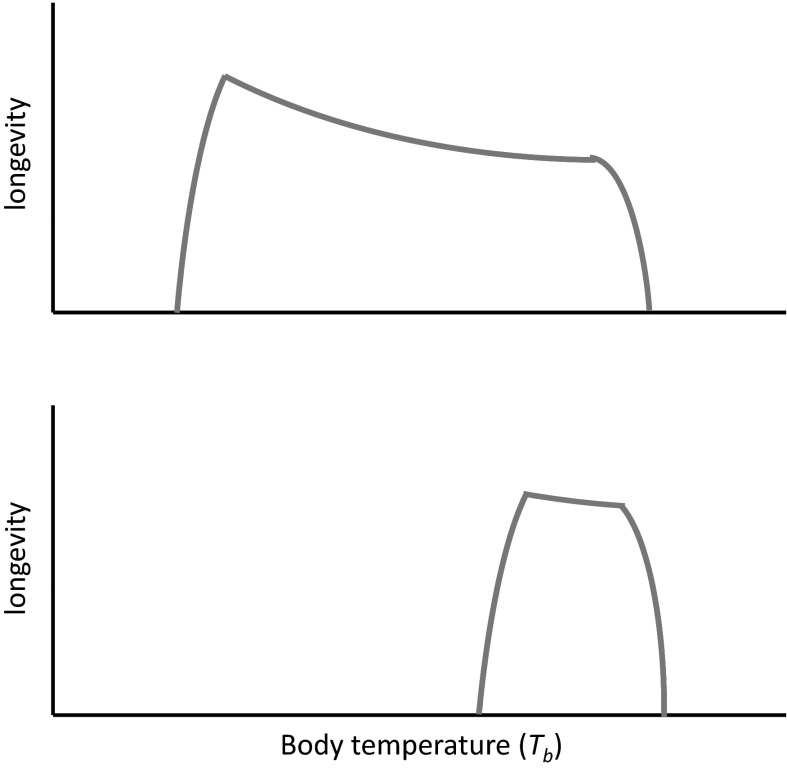
A schematic diagram of predicted body temperature influences on longevity in poikilotherms (*top panel*) and homeotherms (*bottom panel*). Only qualitative differences between poikilotherms and homeotherms are shown, as axes are not quantitative. Away from thermal extremes, longevity predicted by metabolic rate (Gillooly et al. 2001) declines exponentially with increased body temperature

Before exploring thermal effects on ageing and longevity, a distinction needs to be made between the different impacts at thermal extremes compared with *T*_*b*_ within the normal range (Fig. 1). In poikilotherms, very low body temperatures are associated with severe enzyme inhibition (Hochachka and Somero 2002; Portner 2002), leading to a sharp decline in biological function, preventing organismal development. At extreme high temperatures, biological performance also falls off rapidly: problems include insufficient capability of respiratory and circulatory systems to meet increased demand for oxygen (Portner 2002), protein denaturation, and membrane dysfunction due to increased fluidity (Hochachka and Somero 2002). Homeotherms suffer hypo- and hyperthermia when *T*_*b*_ deviates too far below and above the relatively narrow limits of regulated *T*_*b*_. In both poikilotherms and homeotherms, it is the effects of temperature on ageing and longevity across the range of normal *T*_*b*_, rather than under extreme thermal stress, that is the focus of this review.

#### Invertebrates

Early studies focused on the effects of temperature on longevity within invertebrates due to their *T*_*b*_ being determined by their surroundings and thus far easier to manipulate under laboratory conditions than that of homeotherms (Table 1). One of these earlier studies using *Drosophila* *melanogaster* showed that they live about twice as long at 21 °C than at 27 °C (Miquel et al. 1976). Another invertebrate benchmark study by Van Voorhies et al. reported a 75 % increase in lifespan of *C. elegan*s from a 5 °C drop in temperature, consistent between 15–20 and 20–25 °C (Van Voorhies and Ward 1999). Studies by other labs have confirmed these results and it is well-established that temperature has significant effects on the longevity and ageing rate of *Drosophila* and *C. elegan*s (Leiser et al. 2011).

**Table 1:** Longevity effects of temperature manipulation on various animal species

Organism	Temperatures studied (in °C)	% increase in *t*_*0.5*_ (from 5 °C drop unless otherwise specified)	Reference
Invertebrates (model organisms only)			
*D. melanogaster*	18–27	86	(Miquel et al. 1976)
*C. elegans*	15–25	75	(Van Voorhies and Ward 1999)
Poikilothermic vertebrates			
*Cynolebias adloffi*	16 and 22	75 (*t*_*0.45*_; for 6 °C drop)	(Liu and Walford 1966)
*Cynolebias bellottii*	15 and 20	43	(Liu and Walford 1975)
*Nothobranchius furzeri*	22 and 25	14 (for 3 °C drop)	(Valenzano et al. 2006)
*Nothobranchius rachovii*	20–30	57	(Hsu and Chiu 2009)

Strain	Temperature decrease (in °C)	% increase in *t*_*0.5*_	Reference
Mammals (mice)			
Hcrt-UCP2 (females)	0.34	20	(Conti et al. 2006)
Hcrt-UCP2 (males)	0.3	12	

Only laboratory studies specifically manipulating temperature are included. When more than two temperatures were studied, values were averaged. Extreme temperatures in which pathological effects may play a role (McDougall and Mills 1997; Miquel et al. 1976; Van Voorhies and Ward 1999) were excluded from the results

*t*_*0.5*_ is the median lifespan; *t*_*0.45*_ is the age when 45 % of animals have died. See text for details

Many other laboratory studies in insects have reported a negative correlation between temperature and longevity (Gao et al. 2013; Gunay et al. 2010; Kelly et al. 2013; Liu and Tsai 2000; Matadha et al. 2004; Pakyari et al. 2011; Pandey and Tripathi 2008; Wang and Tsai 2001; Zhou et al. 2010). For example, in wasps *Trichogramma platneri* a 5 °C drop in temperature increased median lifespan by 72 % (McDougall and Mills 1997). A large variation of effects of temperature on longevity has been observed in these many studies, though because these species are poorly studied when compared to traditional model organisms husbandry conditions may not always be optimal. Nonetheless, to our knowledge, there is no invertebrate species in which longevity has been shown to increase with temperature, with the exception of pathological effects at very low temperatures. The thermal sensitivities of survival in laboratory studies are supported by studies in the wild, even though these are subject to potentially confounding factors such as the effects of natural enemies on survival (Angilletta et al. 2004).

Low temperature in invertebrates, and ectotherms/poikilotherms in general, is associated with an overall slower life history, including a slower pace of development (Gillooly et al. 2002; Trudgill et al. 2005). Antarctic sponges such as *Cinachyra antarctica* (Epibenthic sponge) and *Scolymastra joubini* (Hexactinellid sponge) are known to have slower growth rates at lower temperatures which may contribute to their exceptional longevity; these are possibly the longest-lived animals on earth, estimated to live up to 1550 and 15,000 years old, respectively (Gatti 2002). This link between temperature and the slowing down of development of ectotherms could possibly support the ‘rate of living hypothesis’ whereby lower temperature promotes longevity by slowing down the rate of reaction of various metabolic processes which affect development and life history. A lower temperature may also reduce damage that is the result of by-products of metabolism such as reactive oxygen species (ROS).

#### Vertebrates (ectotherms)

There have been few controlled studies of the effects of temperature on the longevity of vertebrates. Studies conducted in wild vertebrate populations have found evidence that ectotherms tend to live longer at lower temperatures (Finch 1990; Gosden 1996; Munch and Salinas 2009). This is noticeable in many species of fish (Beverton 1987; Pauly 1980), and for example in the *Sander vitreus* (walleye) and *Cottus bairdii* (mottled sculpin) longevity is dependent on temperature; walleye at lower temperatures in the North USA take longer to grow and reach maturity and live longer than walleye fish in the South (Etnier and Starnes 1993; Finch 1990; Gosden 1996). Overall, although it could be related to extrinsic factors like predation, parasites or infectious diseases unrelated to ageing, there is a clear trend for longer lifespans at lower ambient temperatures in wild species of ectotherms (Munch and Salinas 2009).

In the lab, two classic studies by Walford and colleagues on the effect of temperature on the longevity of the short-lived fish *Cynolebias* showed that a 5 and 6 °C drop in temperature increased lifespan by 43 and 75 %, respectively (Liu and Walford 1966, 1975). Studies on other fishes that gradually senesce, such as *Austrolebias adloffi*, have shown life-extending effects when animals are exposed to lower temperatures (Patnaik et al. 1994). More recent studies in *Nothobranchius furzeri* (Valenzano et al. 2006) showed that a drop from 25 to 22 °C resulted in an increase in lifespan of 14 % (Table 1). In *Nothobranchius rachovii*, the lifespan effects of changes in ambient temperature are reportedly greater with a 50 % increase in lifespan between 30 and 25 °C and a 64 % lifespan increase between 25 and 20 °C (Hsu and Chiu 2009). It should be noted, however, that the survivorship curves in these studies in fish are far from rectangular (and in particular *Nothobranchius* *furzeri* at 22 °C), which is often an indication that other pathological processes apart from ageing are contributing to mortality, perhaps because husbandry conditions are not as optimized in these animals as in more widely used biomedical models, and may be a source of noise. Lastly, late-onset temperature reduction has also been shown to extend lifespan in *Nothobranchius guentheri* (Wang et al. 2014).

Similar results have been observed in amphibians, although from field (rather than laboratory) studies. An intra-species comparison examined the skeletochronology from two datasets: one using two populations (ten urodele and 12 anuran species) and the other dataset using multiple populations (two urodele and nine anuran species), all of which were from separate geographical locations. The study concluded that only the altitude and not latitude gradient correlated with longevity and life history timing (maturation, mean and maximum age) (Zhang and Lu 2012). A possible explanation why the latitude did not correlate with longevity is that there was less variation in temperature along the latitude gradient during the summer (active season for amphibians) in comparison to the altitude gradient (Zhang and Lu 2012). Hypoxia is also thought to reduce the metabolic rate which could be another reason for animals living in oxygen poor regions, i.e. higher altitude regions, living longer (Zhang and Lu 2012).

In *Ambystoma macrodactylum* (Eastern long toed salamander), longevity has been shown to be dependent on temperature with the life history of animals being considerably longer at lower temperatures (Howard 1997). A similar effect has also been observed in the northern species of *Rana aurora* (Californian red-legged frog) in which development appears to be slower and animals probably live longer and lay fewer eggs than its warmer climate counterparts (Davidson 1993). Likewise, in the long-lived olm (*Proteus anguinus*), development is highly temperature-dependent (Bulog and van der Meijden 1992). The current data suggests that, like other poikilotherms, at lower ambient temperatures amphibians have greater longevity, although, because these studies were conducted in the wild, whether this is due to the actual ambient temperature and not to other factors in the field, e.g. less predation, is still open to debate. Nonetheless, these observations support the ‘rate of living hypothesis’ for temperature effects on longevity.

#### Homeotherms

In homeotherms most studies have been conducted in mice. Of note, the Ames dwarf mouse is known to live around 1 year longer than wild-type (Brown-Borg et al. 1996), and it has been speculated that a lower *T*_*b*_ could play a role. In one experiment, six female dwarf mice and six control mice had their *T*_*b*_ measured over 24 h across three separate conditions; ad libitum feeding, food deprivation and cage switching (which caused stress). The results showed that the dwarf mice had a *T*_*b*_ lower than their normal counterparts under all three conditions. For ad libitum feeding the difference was 1.6 °C, with the same difference being maintained during food deprivation which reduced the *T*_*b*_ in both mice. The cage switching caused a rise in *T*_*b*_ for both mice, though dwarf mice still maintained a lower *T*_*b*_ (Hunter et al. 1999).

It has been speculated that the lower *T*_*b*_ of Ames dwarf mice is due to their deficiency in thyroid stimulating hormone (TSH) and growth hormone (GH) with supporting evidence from the Snell dwarf mouse which also has a deficiency in TSH and GH and reduced *T*_*b*_ (Hunter et al. 1999). Ames and Snell mice have mutations in the, respectively, *Prop*-*1* and *Pit*-*1* loci, which lead to a deficiency in TSH, prolactin and GH and lower levels of insulin (Conti 2008). GH receptor (*GHR*) knockout mice and dwarf mice also have lower levels of insulin and circulating insulin-like growth factor 1 (IGF1) (Bartke et al. 2001), similar to CR rodents; as detailed below, CR also lowers *T*_*b*_. Therefore, the insulin pathway alongside GH and TSH is a potential mechanism for body temperature regulation in rodents.

There are many different mutant long-lived mice (Tacutu et al. 2013). Although *T*_*b*_ has only been carefully studied in a small fraction of these, metabolic adjustments and a lower *T*_*b*_ have been observed in some models (Bartke 2011), including the aforementioned Ames and Snell dwarf mice and in *GHR* knockout mice (Hauck et al. 2001). Interestingly, Ames dwarf and *GHR* knockout mice exhibit increased food intake and oxygen consumption per gram of body weight, although it is unknown whether these differences may be due to differences in body weight to surface ratios and/or in body composition (Bartke 2011); that is, increased oxygen consumption could be a compensatory effect to increased heat loss due to increased body surface:mass ratio in these small animals (Bartke and Westbrook 2012). Some mutant long-lived mice also have a normal *T*_*b*_, such as IGF1 receptor heterozygous animals (Holzenberger et al. 2003). Clearly multiple mechanisms are involved in extended longevity, and a lower *T*_*b*_ and alterations in energy metabolism may be included (Bartke and Westbrook 2012), but a lower *T*_*b*_ is not a prerequisite for life-extension.

One landmark study used transgenic mice (Hcrt-UCP2: over-expressed uncoupling protein 2 in hypocretin neurons) to lower the core *T*_*b*_ by 0.3 °C (males) and 0.34 °C (females), resulting in an increase in median lifespan of, respectively, 12 and 20 % (Table 1) (Conti et al. 2006). It should be noted, however, that the mortality curve of control females in Conti et al. (2006) was far from rectangular and they are considerably shorter-lived than males, suggesting potential problems in the experimental conditions. Nonetheless, this study provided causal evidence that a lower *T*_*b*_ increases longevity in mammals. By contrast, one study did not find a correlation between *T*_*b*_ and longevity in individual mice by measuring *T*_*b*_ in female MF1 outbred mice between the ages of 6 and 13 months. Instead, the results suggested that metabolic intensity positively correlated with longevity (Speakman et al. 2004). However, the variance in temperature in this study was not reported and might not have been sufficient for an effect on lifespan to be observed. Although not a model of life-extension, it is also interesting to note that in rats a two-fold and 40 % increase of thyroxin and blood serum triiodothyronine levels, respectively, resulted in a 2 °C increase in *T*_*b*_ as well as a shortening of their lifespan (Bozhkov and Nikitchenko 2014).

In C57B1/6 mice, arguably the most commonly used strain in biogerontology, males tend to live longer than females and several hypotheses have been proposed for this gender longevity difference, such as the unequal inheritance of sex chromosomes, environmental factors and physiology (Sanchez-Alavez et al. 2011). It has also been speculated that *T*_*b*_ could play a role in this gender difference. In C57B1/6 mice, it was shown that young (6 months old) females have a *T*_*b*_ 0.2–0.5 °C higher than their male counterparts. For older (24 months old) mice both genders maintained a similar circadian profile, however at rest the females had a *T*_*b*_ 0.6 °C higher than males. The results also show evidence that the decline of *T*_*b*_ with age may be mediated via reduced locomotor activity. Furthermore, it has been hypothesized that the sex hormones act preferentially on the preoptic area of the hypothalamus which may influence *T*_*b*_ (Sanchez-Alavez et al. 2011). Therefore, gender differences in *T*_*b*_ are influenced by the gonads and are thus perhaps responsible for the differing lifespan between the sexes in C57B1/6 mice (Sanchez-Alavez et al. 2011).

In humans, women appear to have a slightly higher body temperature than men (36.4 ± 0.67 °C (standard error) vs. 36.2 ± 0.61 °C) and mean temperature decreases 0.17 °C between ages 20–30 and ages 70–80 (Waalen and Buxbaum 2011). Another human ageing study, the Baltimore Longitudinal Study of Aging, compared the survival between healthy males in ages ranging from 16 to 95. After age correction, results showed three biomarkers for ageing: temperature, insulin and dehydroepiandrosterone sulphate (DHEAS); lower temperature, insulin and higher DHEAS levels correlated with higher survival rates with no evidence suggesting that any of the individuals underwent CR (Roth et al. 2002). Since *T*_*b*_ declines with age in rodents and in humans, it is also tempting to speculate that this could have, at least indirectly, anti-ageing effects.

### Caloric restriction and temperature

**Fig. 2:**
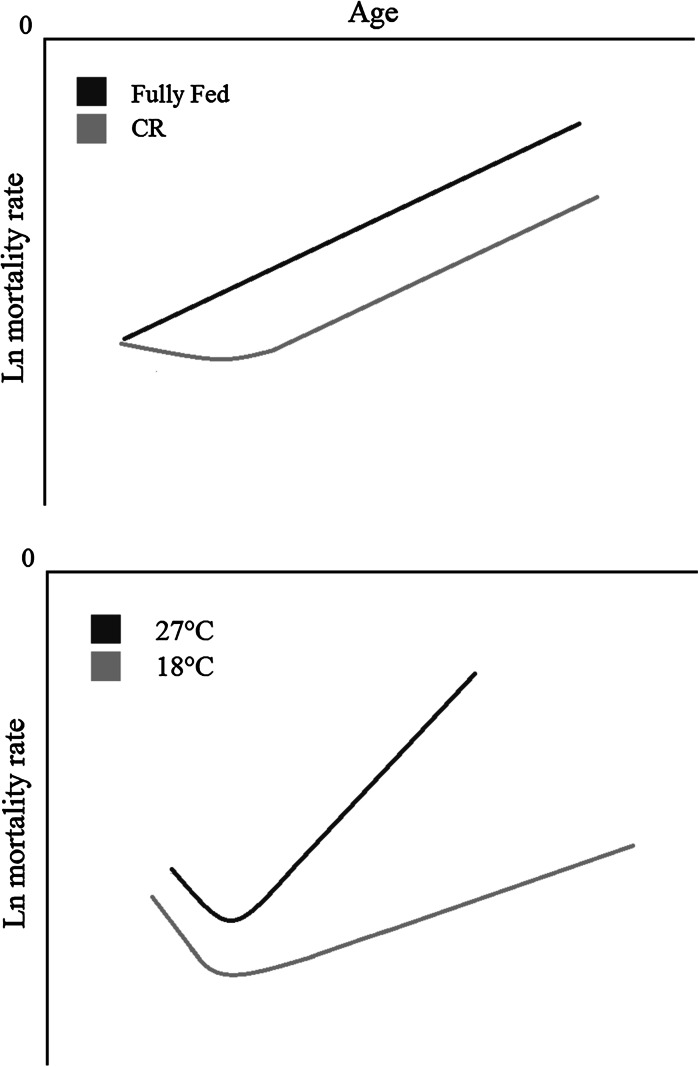
A schematic diagram of the age-specific mortality rates of male *D. melanogaster* which had undergone CR or been fed ad libitum (*top panel*) and at either 27 or 18 °C ambient temperatures (*bottom panel*). Adapted from (Mair et al. 2003)

Caloric restriction, limiting calorie intake without causing malnutrition, has been shown to extend lifespan in multiple model systems (de Magalhaes et al. 2012; Masoro 2005; Spindler 2010). When comparing mortality curve trajectories under CR or reduced *T*_*b*_, however, some studies have observed a difference between the two. Evidence of this comes from *Drosophila* whereby CR initially delays age-related mortality in the short-term, which results in an increase in the overall lifespan, although in the long-term the rate of the mortality trajectory is the same as that of non-CR flies (Fig. 2) (Mair et al. 2003). However, a lowered *T*_*b*_ has a different effect whereby it extends lifespan as well as reducing the slope of the mortality trajectory (Fig. 2) (Miquel et al. 1976), suggesting that in *Drosophila* lowered *T*_*b*_ and CR utilize alternative pathways to reduce mortality. Whether this is the case in homeotherms is unknown.

CR has long been associated with a reduction in *T*_*b*_ with a 1–1.5 °C drop as a general rule in rodents (Duffy et al. 1989; Spindler 2010; Walford and Spindler 1997). Further evidence for CR reducing *T*_*b*_ comes from the LSXSS series of 22 recombinant mouse strains plus six classical inbred strains: under CR, strains such as 12956 and C57BL/6 showed a drop of 1–2 °C in *T*_*b*_ whilst others such as BALB/c and A had a *T*_*b*_ drop of 3–5 °C (Rikke and Johnson 2004). Some other rodent studies have failed to observe a fall in *T*_*b*_ in CR but this may be due to the use of a rectal probe to measure *T*_*b*_ which requires some form of restraint resulting in physiological stress to the rodent which thus may prevent a fall in *T*_*b*_ (Lane et al. 1996). [A more reliable way to measure *T*_*b*_ in rodents by avoiding stress is by using an infrared high performance non-contact thermometer which has been calibrated through implantable micro-chips with temperature transponders (Warn et al. 2003)]. CR in mammals can reduce the metabolic rate and it is believed that it is this reduction which lowers heat production and thus believed to cause a fall in *T*_*b*_. It is thought that this is a mechanism which has evolved to allow animals to cope in times of limited food (Carrillo and Flouris 2011). It should be mentioned, however, that there is some debate concerning the impact of CR on mammalian metabolic rate (Greenberg and Boozer 2000; McCarter and Palmer 1992), and some studies in rodents suggest that CR can extend lifespan without reducing metabolic rate (Masoro 2005).

B6 (lymphoma prone) mice kept at a higher room temperature (30 °C) did not reduce *T*_*b*_ when under CR and this caused a reduction in daily hypothermia and reduced the life-extending effects of CR and in particular its anti-lymphoma action (Koizumi et al. 1996). Likewise, CR is known to reduce cell proliferation and at higher room temperatures this effect weakens. It has therefore been suggested that CR’s induction of hypothermia could cause this anti-lymphoma action which promotes longevity. However, in MRL (autoimmune prone) mice the high room temperature (30 °C) had no effect in reducing the CR-mediated delay of autoimmune diseases (Koizumi et al. 1996).

One emerging important consideration is that CR has strain-specific effects. Surprisingly, when looking across 31 strains with varying CR lifespan effects, reduction in *T*_*b*_ was a negative predictor of life-extension. In other words, strains with a greater *T*_*b*_ reduction were more likely to have shorter lifespans under CR and vice versa. This effect depended on the correlation between temperature and body fat responses, since mouse strains with a lower reduction in *T*_*b*_ under CR also had a lower reduction in body fat, suggesting CR life-extending responses are associated with minimal loss of temperature and least reduction in body fat (Liao et al. 2011). These findings also contradict the aforementioned results on Hcrt-UCP2 mice that suggested that a modest reduction in *T*_*b*_ has marked effects on longevity by itself, which is clearly not observed in theses strains. That said, these studies may not be directly comparable since one previous study on the same strains found that differences in *T*_*b*_ were associated with variation in the rate of heat loss, not heat production (Rikke and Johnson 2007). All in all, it does not appear that the longevity effects of CR in mice are *per se* a result of a drop in *T*_*b*_.

Studies are also emerging concerning the effects of CR on rhesus monkeys, suggesting health benefits but more modest (if any) lifespan effects than observed in rodents (Colman et al. 2009; Mattison et al. 2012). In rhesus monkeys placed under CR for 6 years it was shown that a fall in 30 % of calorie intake reduced the *T*_*b*_ by 0.5 °C compared to ad libitum feeding after matching for age. In 2.5 year-old monkeys on a short-term (1 month) CR of 30 %, the *T*_*b*_ drop was 1.0 °C. For short-term CR the energy expenditure had fallen by 24 % over 24 h. The association between a fall in *T*_*b*_ and energy expenditure suggests that CR acts to conserve energy by reducing the *T*_*b*_. In the long-term study, however, temperature was only measured in the mid-morning when it may have been more reliable to record it throughout the day (Lane et al. 1996). Another study in rhesus monkeys also showed that CR caused roughly a 0.7 °C drop in *T*_*b*_ as well as reductions in insulin and higher serum DHEAS (Roth et al. 2002).

A CR study in humans (Soare et al. 2011) used three groups each consisting of 24 individuals; one group on a CR diet for 6 years, another consisting of endurance runners (EX) and the final group of healthy individuals with a sedentary lifestyle (less than 1 h of exercise per week) who ate a western diet (WD). For the CR group, their energy intake was 23 and 37 % lower than the WD and EX groups, respectively. All groups were matched according to age and sex, with the EX group being matched on body fat percentage to the CR group. Each individual then had their *T*_*b*_ measured every minute over a 24-h period using an ingested telemetric capsule. The results showed that *T*_*b*_ was lowest amongst the CR group with a mean 24-h temperature of 36.64 °C ± 0.16 (SD) in comparison to 36.86 °C ± 0.2 for EX and 36.83 °C for WD. It was discovered that the percentage of body fat is also linked to 24-h *T*_*b*_, although body fat is not likely to play a role in *T*_*b*_ reduction as both the EX and CR groups had a similar low percentage of body fat, however the mean 24-h, day-time and night-time *T*_*b*_ of the CR group was roughly 0.2 °C lower than that of the EX group. Therefore, *T*_*b*_ was thought to be controlled primarily through CR (Soare et al. 2011).

CR responses can be thought of as an energy conservation mechanism which, over the long-term in monkeys, humans and rodents, can cause a fall in circulating triiodothyronine levels which regulate body temperature and thus causes the *T*_*b*_ to drop (Soare et al. 2011). Short-term CR also reduces *T*_*b*_ in overweight individuals attempting to lose weight, however in overweight, weight stable individuals this was not shown to be the case. In normal and obese individuals with stable weight, *T*_*b*_ remains the same in both; obese individuals are thought to achieve this via heat dissipation in their more peripheral regions. CR individuals also have lower levels of insulin, leptin and total testosterone which may also contribute to heat reduction (Soare et al. 2011). Another study conducted using human volunteers who underwent CR (25 % CR of the baseline energy requirement) for 6 months exhibited lower levels of fasting serum insulin, a significant fall in both the total 24-h and sleeping energy expenditure, which correlated with a drop in thyroid hormone concentration, as well as a 0.2 °C decrease in *T*_*b*_. Similar results were also shown in their CREX group (12 % CR and 12 % increase in energy expenditure) including a drop of 0.3 °C in *T*_*b*_. Both the CR, CREX and the very low calorie diet group (890 kcal/d) had lower levels of DNA damage suggesting a possible mechanism for promoting longevity, however whether this is maintained long-term has yet to be verified (Heilbronn et al. 2006).

### Mechanisms of temperature life-extending effects

Thermodynamic explanations to ageing have been long proposed, in which ageing is the result of an increase in molecular disorder and a decrease in metabolic stability (Demetrius 2013). The intuitive interpretation for how low body temperature extends longevity is that it affects metabolic rates and this decreases the rate of biochemical reactions and retards whatever process(es) cause(s) ageing. Since metabolic rate increases exponentially with temperature (Gillooly et al. 2001), it is reasonable to postulate that temperature acts exponentially on 1/lifespan and hence that lower temperature acts exponentially to extend lifespan (Fig. 1), especially over a small temperature range, and indeed this has been shown in some studies in poikilotherms (McDougall and Mills 1997; Munch and Salinas 2009). One study in the housefly (*Musca domestica*) supported this idea by showing that animals at 15 °C lived longer but were much less active than at 23 °C, and that lower physical activity by itself was associated with a longer lifespan (Ragland and Sohal 1975). Inducing mild heat stress can also increase the lifespan in both *D. melanogaster* and *C. elegans* by stimulating pathways associated with genome stability in an effect known as hormesis. A recent study which heat shocked (34 °C for 2 h) younger male *D. melanogaster* three times showed an up-regulation of genes involved in the cellular response to heat 10–51 days after heat shocking, suggesting that the heat shock HSP70 pathway may be involved in fly longevity (Sarup et al. 2014). Although the specific mechanisms for the long lifespan of mice with a lower core *T*_*b*_ remain unknown, an increase in energy efficiency was observed that appears to be related to the reduced metabolic requirements to maintain a lower *T*_*b*_ (Conti et al. 2006). This in turn could impact on various forms of molecular damage, including oxidative stress and DNA damage (Farmer and Sohal 1987; Lindahl and Nyberg 1972).

The fact that CR does not always extend lifespan, and in diverse mouse strains its longevity effect is actually associated with smaller decreases in temperature, suggests that other factors may obscure or reverse expected effects of *T*_*b*_ on ageing. That women have higher temperatures than men and yet live longer, while female C57B1/6 mice have higher temperatures than males and live less, supports this. Similarly, it has long been argued that a drop in *T*_*b*_ reduces the production of ROS which then prolongs lifespan (Conti 2008). A reduction in oxidative damage and enhanced antioxidant systems in ectothermic animals kept at lower temperatures (and thus longer-lived) has been observed, for example in fish (Hsu and Chiu 2009; Wang et al. 2014). More recently, however, the free radical theory of ageing has come under fire due to a plethora of studies with discouraging results, raising questions about the role of ROS in ageing (reviewed in de Magalhaes and Church 2006; Lapointe and Hekimi 2010). Therefore, it appears that the view that a drop in *T*_*b*_ by itself increases lifespan is overly simplistic.

More recent results suggesting that specific mechanisms retard ageing in response to lower temperature challenge thermodynamic explanations. In *C. elegans* it has been discovered that its cold sensing TRP channel TRPA-1 can promote longevity when there is a drop in temperature by inducing a calcium influx into the cell, activating PKC which via SGK-1 activates the DAF-16/FOXO transcription factor (Xiao et al. 2013). *Caenorhabditis elegans* thermosensory neurons can also play a role in the effect temperature has on longevity at warmer temperatures (25 °C). Mutations in thermosensory neurons have been shown to shorten lifespan even further in warmer temperatures by causing a fall in the expression levels of *daf*-*9*. DAF-9 regulates the nuclear hormone receptor DAF-12 and so *daf*-*12* null mutations can inhibit this process (Lee and Kenyon 2009). Overall, these results demonstrate that the temperature effect on *C. elegans* longevity is not a passive process and further challenges the ‘rate of living theory’ (Xiao et al. 2013). Similarly, in fruit flies, changing back and forth the ambient temperature from hot to cold, animals have a similar longevity of flies kept in cold conditions (Liu and Walford 1972; Rikke and Johnson 2004), which suggest that it is not the exposure time to cold but rather physiological adaptations to it that are important.

It has been proposed that CR reduces *T*_*b*_ which in turn affects the hormonal axis that may have downstream targets that increase lifespan (Mobbs et al. 2001). An endocrine role thus seems plausible. Temperature in ectotherms can act on hormones associated with growth and development to induce heterochrony (McKinney and McNamara 1991). Indeed, there is evidence from various studies suggesting that the GH/IGF system mediates the effects of temperature on the growth and development of fish (reviewed in Gabillard et al. 2005). Interestingly, one study of exercise in young men showed a strong relationship between *T*_*b*_ increase during exercise and GH levels, although this was not dependent on ambient temperature; this suggests that body temperature could in some way be a stimulus for GH secretion (Bridge et al. 2003). Recall, however, that we previously mentioned that GH/insulin is also a possible mechanism for *T*_*b*_ regulation, and thus the causality of these events remains to be fully elucidated.

Another hypothesis is that a lower *T*_*b*_ promotes longevity through utilizing various metabolic pathways which suppress autoimmunity in old age (Rikke and Johnson 2004). A lower *T*_*b*_ also increases resistance to environmental factors, which has been shown in hypothermic rodents to have a greater resistance to irradiation. This is further highlighted in hypothermic rabbits which had a 10× greater resistance to bacterial endotoxins than normal rabbits (Liu and Walford 1972; Rikke and Johnson 2004). A lower developmental temperature in *D. melanogaster* also results in enhanced stress resistance (Kim et al. 2010), at least to some types of stress.

In terms of responses to lower temperature in homeotherms, brown adipose tissue (BAT), which contains large amounts of mitochondria, is used to generate heat in cold environments via uncoupling proteins, in particular UCP1 within the mitochondrial membrane. Mice deficient in UCP1 have been shown to have an increased susceptibility to obesity during ageing when exposed to a high fibre diet and kept at a constant room temperature of 23 °C (Kontani et al. 2005). In humans, the A-3826G polymorphism in the *UCP1* gene promoter region reduces UCP1 expression and could be more susceptible to a higher body mass including greater levels of subcutaneous fat (Sramkova et al. 2007). Another role which has been established for UCP is in the reduction of ROS production when up-regulated and it has been proposed that this is one of the factors which might contribute to the link between lower *T*_*b*_ and longevity (Carrillo and Flouris 2011). In rats exposed to the cold or treated with triiodothyronine, UCP3 was up-regulated in BAT, suggesting that UCP3 has a role in the regulation of *T*_*b*_ and energy expenditure (Larkin et al. 1997). Research using 6 month-old 344 Fischer rats showed a 4-fold increase of UCP1, a 2-fold increase of interscapular BAT mass and a 26-fold increase in cell proliferation after cold exposure, whereas rats at 26 months showed no such increase. This further shows that the response to temperature varies depending on age (Florez-Duquet et al. 1998). Age-related thermogenic impairment possibly contributes to diabetes and obesity in ageing, and recent results from mice suggest it may be mediated by ghrelin signalling (Lin et al. 2014). Moreover, BAT activity and mass decline with age, even if this is much more pronounced in men than in women (Pfannenberg et al. 2010). Lastly, when exposed to cold temperature, mice tend to have lower levels of body fat than mice in surroundings with normal temperature levels, this reduction in body fat by a fall in temperature has been thought to be the reason for a fall in tumour incidences (Carrillo and Flouris 2011), in particular given the known links between obesity and cancer, including in an age-related fashion (de Magalhaes 2013).

Although it is possible that a low temperature decreases accumulation of molecular damage, this appears to be an oversimplistic interpretation, in particular in mammals in which various physiological processes, such as in adipose tissue and neuroendocrine changes, are associated with temperature and ageing. More integrative studies of the relationships between temperature, ageing mechanisms and longevity are warranted.

### Species correlations between temperature and longevity

In homeotherms undergoing torpor or hibernation, *T*_*b*_ falls and endothermy is temporarily replaced with ectothermy. Hibernation can also increase the survival rate by five times between similar-sized hibernating and non-hibernating mammals (Turbill et al. 2011). Indeed, a study using a super tree (50 % of longevity records from wild population studies where mortality is not controlled) of Chiroptera showed that bats’ lifespan correlates strongly with the length of hibernation, body mass and occasional cave use (Wilkinson and South 2002). Another study found that smaller mammals which hibernate tend to have longer maximum lifespans (50 % greater for 50 g of species), lower reproductive rates and longer generation times than similar-sized non-hibernating species (Turbill et al. 2011).

Hibernation protects against predation by reducing the chance of detection via lowering levels of metabolism, having a cold and motionless body and lower emissions of body odours. As such, it is thought that predator avoidance is the main reason behind hibernation in small mammals rather than the conservation of energy, as hibernation can occur during times of the year when sufficient food is available (Turbill et al. 2011). It has been proposed that insulin can cause hypothermia during hibernation, although this remains unproven, but it can act independently of lower *T*_*b*_ as shown in studies using poikilotherms (Andrews 2007). Moreover, hibernation could be used in times of limited food supply and hence have a similar role as CR. In bats, hibernation reduces *T*_*b*_ by 85 % (from 40 to 6 °C) which is then maintained over a course of several weeks at a time. This reduces the metabolic rate to 5 % of the room temperature resting rate which reduces the amount of energy used by the animal; thus it might reduce the oxidative damage caused by internal metabolic pathways and increase lifespan (Wilkinson and South 2002), although as discussed above this simplistic interpretation has been attacked.

Comparisons across species have largely disproved the rate of living theory in homeotherms (de Magalhaes et al. 2007; Speakman 2005). A low *T*_*b*_ is by no means a prerequisite for the evolution of longevity or always associated with it. For example, birds tend to have a higher *T*_*b*_ and live longer than similar-sized mammals; likewise, marsupials have a low *T*_*b*_ when compared to placental mammals and generally have shorter lifespans (de Magalhaes et al. 2007). A large comparative study of temperature, metabolic rate, body size and longevity in mammals revealed a strong positive correlation between *T*_*b*_ and basal metabolic rate (BMR), yet no correlation between BMR and longevity. However, a negative correlation was observed between *T*_*b*_ and time to maturity and a weak, borderline significant negative correlation was also observed between *T*_*b*_ and longevity (de Magalhaes et al. 2007). Although other factors, like ecological constraints (Demetrius and Gundlach 2014), are involved in the evolution of life histories and mammals have a very narrow range of body temperatures, these results suggest that *T*_*b*_ might affect the timing of life history events in ways unrelated to metabolic rate.

#### Influence of temperature on the long lifespan of the naked mole-rat

Capable of living over 30 years (Tacutu et al. 2013), the naked mole-rat (*Heterocephalus glaber*) is the longest-lived rodent and is exceptionally resistant to cancer (Buffenstein 2008). This has led to a growing number of studies in this species (Gorbunova et al. 2014), including large-scale genome sequencing efforts (Keane et al. 2014). Naked mole-rats are also unique among mammals in that they are poikilotherms and have a low metabolic rate and body temperature when compared to other mammals (Buffenstein and Yahav 1991; McNab 1980). Therefore, and given the aforementioned impact of temperature on longevity, one major open question is whether the exceptional longevity of the naked mole-rat could at least partly be due to its comparatively low *T*_*b*_. We attempted to explore this by extrapolating from other studies.

Wild-derived mice, as these are more likely to be representative of the *Mus musculus* species than laboratory strains, have a *T*_*b*_ of 36.9 °C (Tacutu et al. 2013). Because naked mole-rats are incapable of maintaining their body temperature, their average *T*_*b*_ during their lifespan is not known for sure; the AnAge database of ageing and longevity in animals lists 32.1 °C, taken from (White and Seymour 2003), and one study reported a mean *T*_*b*_ of 30.6 °C (McNab 1980). However, pregnant females have a higher *T*_*b*_ by ~1.5 °C (Buffenstein et al. 1996), and yet there is no evidence that queens have a shorter lifespan (Buffenstein 2008). Because a female can become a breeding queen early in life, and can essentially be pregnant most of the time, *T*_*b*_ of breeding females can be estimated based on the *T*_*b*_ increase during the various stages of pregnancy (Buffenstein et al. 1996), and assuming that a female can be pregnant continuously with only a one-week interval between pregnancies, since gestation in naked mole-rats is ~10 weeks with as little as 1 week post-partum estrus (Buffenstein et al. 1996). Mean estimated *T*_*b*_ is thus (1 week × 30 °C + 3 × 33.4 + 4 × 34.6 + 3 × 34. 8)/11 = 34.2 °C. As this assumes continuous pregnancies throughout adult lifespan, 34.2 °C marks an upper limit for *T*_*b*_, with the lower value of 32.1 °C a realistic range of mean *T*_*b*_ values; the data from White and Seymour (2003) is also based on multiple studies and thus preferred to McNab (1980).

Given that the results from various animals show that a lower *T*_*b*_ increases longevity, it seems plausible that the low *T*_*b*_ of naked mole-rats contributes to their longevity. Although non-contentious and clearly important, this issue has not been addressed before. Therefore, we attempted to estimate quantitatively the effect of *T*_*b*_ on the longevity of the naked mole-rat based on studies conducted on other organisms. Specifically, we employed those organisms on Table 1, with the exception of transgenic mice given that the magnitude of changes observed are inconsistent with the abovementioned results from other mouse strains and with the thermal sensitivity of longevity from species comparisons (Table 2).

**Table 2:** Longevity and body temperature of mole-rats and selected related species

Species	Family	*t*_*max*_ (years)	Body weight (g)	*T*_*b*_ (°C)
Mole-rats				
*Cryptomys anselli*	Bathyergidae	20.6	90	
*Cryptomys damarensis*	Bathyergidae	15.5	180	35.2
*Cryptomys hottentotus*	Bathyergidae	11^a^	132.5	34.4
*Cryptomys mechowi*	Bathyergidae	16^a^	250	34
*Georychus capensis*	Bathyergidae	11.2^a^	181	36.4
*Heliophobius argenteocinereus*	Bathyergidae		160	35.1
*Heterocephalus glaber*	Bathyergidae	31	35	32.1
*Nannospalax ehrenbergi*	Muridae	20.2	160	35.5
Other rodents				
*Cavia porcellus*	Caviidae	12	728	39
*Mus musculus*	Muridae	4	20.5	36.9
*Rattus norvegicus*	Muridae	3.8	300	37.1
*Sciurus carolinensis*	Sciuridae	23.6	533	38.7
Average for rodents^b^		9.2	1175	36.8

^a^Species with questionable longevity records that may be significantly underestimated

^b^Only rodents for which data on body temperature (*T*_*b*_) and maximum lifespan (*t*_*max*_) is available in the AnAge database (Tacutu et al. 2013) were included

To extrapolate *T*_*b*_ effects on longevity from studies in various species, a linear regression of ln-transformed lifespan against temperature produced a slope from which the longevity of mice with the *T*_*b*_ of naked mole-rats was predicted. The use of semi-log plots of lifespan against temperature is based on the aforementioned assumption that temperature acts exponentially on 1/lifespan, in line with previous studies (McDougall and Mills 1997; Munch and Salinas 2009). Given the effects of temperature on the longevity of various species, the longevity of mice at the *T*_*b*_ of naked mole-rats (32.1–34.2 °C) can be estimated as 4.5–7.3 years. The lowest predicted effect of temperature on longevity is the result from *Nothobranchius* *furzeri* (Table 1), for which we feel further studies are necessary given the aforementioned concerns regarding the survival curve at the lower temperature. Excluding *Nothobranchius* *furzeri*, and averaging the other species, we estimate that hypothetically mice with the body temperature of the naked mole-rat may live 30–65 % longer or 5.3–6.6 years. It is also tempting to hypothesize that, assuming these calculations can be extrapolated to them, naked mole-rats with a *T*_*b*_ of 37 °C might not live more than 20–25 years. Our approach, of course, assumes that intra-species effects can be extrapolated across species and is thus only a first approximation of the contribution of *T*_*b*_ to the longevity of naked mole-rats.

Our view is that body temperature likely explains a small part of the exceptional longevity of the naked mole-rat. We also think that consideration should be given to body temperature in comparative studies of ageing. Mole-rats in general have a low *T*_*b*_ in species comparisons (Table 2), possibly because they live in stable, protected environments, and it is tempting to speculate that this has contributed to the evolution of longevity in mole-rats of different taxa. Not surprisingly, the naked mole-rat has the longest lifespan and the lowest *T*_*b*_ of studied mole-rats (Table 2). In this context, other small, long-lived rodents like the Eastern gray squirrel (*Sciurus carolinensis*), which can live up to 23.6 years and actually has a higher *T*_*b*_ (38.7 °C) than the rodent average (Table 2), may be equally informative to biogerontology. On the other hand, the bowhead whale (*Balaena mysticetus*) is the longest-lived mammal (estimated maximum lifespan of 211 years), which has led to its genome being recently sequenced (Keane et al. 2015). Its average *T*_*b*_ has been estimated to be 33.8 °C (SD = 0.83, N = 28) which is lower than other non-hibernating eutherian mammals, and its metabolic resting rate also appears to be lower than that of other cetaceans (George 2009). While additional studies are warranted, a relatively low *T*_*b*_ might have contributed, even if to a small degree, to the bowhead’s exceptional longevity. We hope these considerations, although speculative, will stimulate further work on the important topic of temperature effects on species longevity.

## Concluding remarks

It is clear that temperature affects longevity from invertebrates to mammals both within species and possibly across species. This was initially thought to be due to thermodynamic effects and the direct effects of low metabolism (e.g., reduced oxidative and/or DNA damage), but now a picture is emerging that, as often happens in biology, the mechanisms are not as simple as once thought. The interplay of processes involved suggests a role of neuroendocrine mechanisms in responses to low temperature that in turn impact on ageing and longevity.

## References

[CR1] Andrews MT (2007). Advances in molecular biology of hibernation in mammals. BioEssays.

[CR2] Angilletta MJ, Steury TD, Sears MW (2004). Temperature, growth rate, and body size in ectotherms: fitting pieces of a life-history puzzle. Integr Comp Biol.

[CR3] Bartke A (2011). Growth hormone, insulin and aging: the benefits of endocrine defects. Exp Gerontol.

[CR4] Bartke A, Westbrook R (2012). Metabolic characteristics of long-lived mice. Front Genet.

[CR5] Bartke A, Coschigano K, Kopchick J, Chandrashekar V, Mattison J, Kinney B, Hauck S (2001). Genes that prolong life: relationships of growth hormone and growth to aging and life span. J Gerontol Ser A Biol Sci Med Sci.

[CR6] Beverton RJH, Woodhead AD, Beverton RJH (1987). Longevity in fish: some ecological and evolutionary considerations. Evolution in animals.

[CR7] Bozhkov A, Nikitchenko YV (2014). Thermogenesis and longevity in mammals. Thyroxin model of accelerated aging. Exp Gerontol.

[CR8] Bridge MW, Weller AS, Rayson M, Jones DA (2003). Ambient temperature and the pituitary hormone responses to exercise in humans. Exp Physiol.

[CR9] Brown-Borg HM, Borg KE, Meliska CJ, Bartke A (1996). Dwarf mice and the ageing process. Nature.

[CR10] Buffenstein R (2008). Negligible senescence in the longest living rodent, the naked mole-rat: insights from a successfully aging species. J Comp Physiol B.

[CR11] Buffenstein R, Yahav S (1991). Is the naked mole-rat *Heterocephalus glaber* a poikilothermic or poorly thermoregulating endothermic mammal?. J Therm Biol.

[CR12] Buffenstein R, Urison NT, Woodley R, van der Westhuizen LA, Jarvis J (1996). Temperature changes during pregnancy in the subterranean naked mole-rat (*Heterocephalus glaber*): the role of altered body composition and basking behaviour. Mammalia.

[CR13] Bulog B, van der Meijden A (1992) Proteus anguinus. http://amphibiaweb.org/

[CR14] Carrillo AE, Flouris AD (2011). Caloric restriction and longevity: effects of reduced body temperature. Ageing Res Rev.

[CR15] Colman RJ (2009). Caloric restriction delays disease onset and mortality in rhesus monkeys. Science.

[CR16] Conti B (2008). Considerations on temperature, longevity and aging. Cell Mol Life Sci.

[CR17] Conti B (2006). Transgenic mice with a reduced core body temperature have an increased life span. Science.

[CR18] Davidson C (1993) Californian nevada amphibian population task force. http://www.canvamphibs.com/Species/aurora.html

[CR19] de Magalhaes JP (2013). How ageing processes influence cancer. Nat Rev Cancer.

[CR20] de Magalhaes JP, Church GM (2006). Cells discover fire: employing reactive oxygen species in development and consequences for aging. Exp Gerontol.

[CR21] de Magalhaes JP, Costa J, Church GM (2007). An analysis of the relationship between metabolism, developmental schedules, and longevity using phylogenetic independent contrasts. J Gerontol Ser A Biol Sci Med Sci.

[CR22] de Magalhaes JP, Wuttke D, Wood SH, Plank M, Vora C (2012). Genome-environment interactions that modulate aging: powerful targets for drug discovery. Pharmacol Rev.

[CR23] Demetrius LA (2013). Boltzmann, Darwin and directionality theory. Phys Rep.

[CR24] Demetrius LA, Gundlach VM (2014). Directionality theory and the entropic principle of natural selection. Entropy Switz.

[CR25] Duffy PH, Feuers RJ, Leakey JA, Nakamura K, Turturro A, Hart RW (1989). Effect of chronic caloric restriction on physiological variables related to energy metabolism in the male Fischer 344 rat. Mech Ageing Dev.

[CR26] Etnier DA, Starnes WC (1993). The fishes of Tennessee.

[CR27] Farmer KJ, Sohal RS (1987). Effects of ambient temperature on free radical generation, antioxidant defenses and life span in the adult housefly, *Musca domestica*. Exp Gerontol.

[CR28] Finch CE (1990). Longevity, senescence, and the genome.

[CR29] Florez-Duquet M, Horwitz BA, McDonald RB (1998). Cellular proliferation and UCP content in brown adipose tissue of cold-exposed aging Fischer 344 rats. Am J Physiol Regul Integr Comp Physiol.

[CR30] Gabillard JC, Weil C, Rescan PY, Navarro I, Gutierrez J, Le Bail PI (2005). Does the GH/IGF system mediate the effect of water temperature on fish growth? A review. Cybium.

[CR31] Gao G-Z, Perkins LE, Zalucki MP, Lu Z-Z, Ma J-H (2013). Effect of temperature on the biology of Acyrthosiphon gossypii Mordvilko (Homoptera: Aphididae) on cotton. J Pest Sci.

[CR32] Gatti S (2002) The role of sponges in high-Antarctic carbon and silicon cycling: a modelling approach. Alfred-Wegener-Institut für Polar-und Meeresforschung, Bremerhaven

[CR33] George JC (2009) Growth, morphology and energetics of bowhead whales (*Balaena mysticetus*). University of Alaska Fairbanks, Fairbanks

[CR34] Gillooly JF, Brown JH, West GB, Savage VM, Charnov EL (2001). Effects of size and temperature on metabolic rate. Science.

[CR35] Gillooly JF, Charnov EL, West GB, Savage VM, Brown JH (2002). Effects of size and temperature on developmental time. Nature.

[CR36] Gorbunova V, Seluanov A, Zhang Z, Gladyshev VN, Vijg J (2014). Comparative genetics of longevity and cancer: insights from long-lived rodents. Nat Rev Genet.

[CR37] Gosden R (1996). Cheating time.

[CR38] Greenberg JA, Boozer CN (2000). Metabolic mass, metabolic rate, caloric restriction, and aging in male Fischer 344 rats. Mech Ageing Dev.

[CR39] Gunay F, Alten B, Ozsoy ED (2010). Estimating reaction norms for predictive population parameters, age specific mortality, and mean longevity in temperature-dependent cohorts of *Culex quinquefasciatus* Say (Diptera: Culicidae). J Vector Ecol.

[CR40] Hauck SJ, Hunter WS, Danilovich N, Kopchick JJ, Bartke A (2001). Reduced levels of thyroid hormones, insulin, and glucose, and lower body core temperature in the growth hormone receptor/binding protein knockout mouse. Exp Biol Med (Maywood).

[CR41] Hayflick L (2007). Biological aging is no longer an unsolved problem. Ann N Y Acad Sci.

[CR42] Heilbronn LK (2006). Effect of 6-month calorie restriction on biomarkers of longevity, metabolic adaptation, and oxidative stress in overweight individuals: a randomized controlled trial. JAMA.

[CR43] Hochachka PW, Somero GN (2002). Biochemical adaptation: mechanism and process in physiological evolution.

[CR44] Holzenberger M (2003). IGF-1 receptor regulates lifespan and resistance to oxidative stress in mice. Nature.

[CR45] Hsu CY, Chiu YC (2009). Ambient temperature influences aging in an annual fish (*Nothobranchius rachovii*). Aging Cell.

[CR46] Hunter W, Croson W, Bartke A, Gentry M, Meliska C (1999). Low body temperature in long-lived Ames dwarf mice at rest and during stress. Physiol Behav.

[CR47] Howard JL (1997) *Ambystoma macrodactylum* In: Fire effects information system. http://www.fs.fed.us/database/feis/

[CR48] Keane M (2014). The naked mole rat genome resource: facilitating analyses of cancer and longevity-related adaptations. Bioinformatics.

[CR49] Keane M (2015). Insights into the evolution of longevity from the bowhead whale genome. Cell reports.

[CR50] Kelly MA, Zieba AP, Buttemer WA, Hulbert AJ (2013). Effect of temperature on the rate of ageing: an experimental study of the blowfly *Calliphora stygia*. PLoS One.

[CR51] Kim K, Lin YR, Park Y (2010). Enhancement of stress resistances and downregulation of Imd pathway by lower developmental temperature in *Drosophila melanogaster*. Exp Gerontol.

[CR52] Koizumi A (1996). A tumor preventive effect of dietary restriction is antagonized by a high housing temperature through deprivation of torpor. Mech Ageing Dev.

[CR53] Kontani Y (2005). UCP1 deficiency increases susceptibility to diet-induced obesity with age. Aging Cell.

[CR54] Lane MA (1996). Calorie restriction lowers body temperature in rhesus monkeys, consistent with a postulated anti-aging mechanism in rodents. Proc Natl Acad Sci.

[CR55] Lapointe J, Hekimi S (2010). When a theory of aging ages badly. Cell Mol Life Sci.

[CR56] Larkin S (1997). Regulation of the third member of the uncoupling protein family, UCP3, by cold and thyroid hormone. Biochem Biophys Res Commun.

[CR57] Lee S-J, Kenyon C (2009). Regulation of the longevity response to temperature by thermosensory neurons in *Caenorhabditis elegans*. Curr Biol.

[CR58] Leiser SF, Begun A, Kaeberlein M (2011). HIF-1 modulates longevity and healthspan in a temperature-dependent manner. Aging Cell.

[CR59] Liao CY, Rikke BA, Johnson TE, Gelfond JA, Diaz V, Nelson JF (2011). Fat maintenance is a predictor of the murine lifespan response to dietary restriction. Aging Cell.

[CR60] Lin L, Lee JH, Bongmba OY, Ma X, Zhu X, Sheikh-Hamad D, Sun Y (2014). The suppression of ghrelin signaling mitigates age-associated thermogenic impairment. Aging Albany N Y.

[CR61] Lindahl T, Nyberg B (1972). Rate of depurination of native deoxyribonucleic acid. Biochemistry.

[CR62] Liu YH, Tsai JH (2000). Effects of temperature on biology and life table parameters of the Asian citrus psyllid, *Diaphorina citri* Kuwayama (Homoptera : Psyllidae). Ann Appl Biol.

[CR63] Liu RK, Walford RL (1966). Increased growth and life-span with lowered ambient temperature in the annual fish *Cynolebias adloffi*. Nature.

[CR64] Liu RK, Walford RL (1972). The effect of lowered body temperature on lifespan and immune and non-immune processes. Gerontologia.

[CR65] Liu RK, Walford RL (1975). Mid-life temperature-transfer effects on life-span of annual fish. J Gerontol.

[CR66] Loeb J, Northrop JH (1916). Is there a temperature coefficient for the duration of life?. Proc Natl Acad Sci USA.

[CR67] MacArthur J, Baillie W (1929). Metabolic activity and duration of life. I. Influence of temperature on longevity in *Daphnia magna*. J Exp Zool.

[CR68] Mair W, Goymer P, Pletcher SD, Partridge L (2003). Demography of dietary restriction and death in *Drosophila*. Science.

[CR69] Masoro EJ (2005). Overview of caloric restriction and ageing. Mech Ageing Dev.

[CR70] Matadha D, Hamilton GC, Lashomb JH (2004) Effect of temperature on development, fecundity, and life table parameters of *Encarsia citrina* Craw (Hymenoptera: Aphelinidae), a parasitoid of *Euonymus scale*, *Unaspis euonymi* (Comstock), and *Quadraspidiotus perniciosus* (Comstock) (Homoptera: Diaspididae). Environ Entomol 33:1185–1191

[CR71] Mattison JA (2012). Impact of caloric restriction on health and survival in rhesus monkeys from the NIA study. Nature.

[CR72] McCarter RJ, Palmer J (1992). Energy metabolism and aging: a lifelong study of Fischer 344 rats. Am J Physiol.

[CR73] McDougall SJ, Mills NJ (1997). The influence of hosts, temperature and food sources on the longevity of *Trichogramma platneri*. Entomol Exp Appl.

[CR74] McKinney ML, McNamara KJ (1991). Heterochrony: the evolution of ontogeny.

[CR75] McNab BK (1980). On estimating thermal conductance in endotherms. Physiol Zool.

[CR76] Miquel J, Lundgren PR, Bensch KG, Atlan H (1976). Effects of temperature on the life span, vitality and fine structure of *Drosophila melanogaster*. Mech Ageing Dev.

[CR77] Mobbs CV (2001). Neuroendocrine and pharmacological manipulations to assess how caloric restriction increases life span. J Gerontol Ser A.

[CR78] Munch SB, Salinas S (2009). Latitudinal variation in lifespan within species is explained by the metabolic theory of ecology. Proc Natl Acad Sci USA.

[CR79] Pakyari H, Fathipour Y, Enkegaard A (2011). Effect of temperature on life table parameters of predatory thrips *Scolothrips longicornis* (Thysanoptera: Thripidae) fed on twospotted spider mites (Acari: Tetranychidae). J Econ Entomol.

[CR80] Pandey AK, Tripathi CPM (2008) Effect of temperature on the development, fecundity, progeny sex ratio and life-table of *Campoletis chlorideae*, an endolarval parasitoid of the pod borer, *Helicoverpa armigera*. Biocontrol 53:461–471. doi:10.1007/s10526-007-9083-3

[CR81] Patnaik B, Mahapatro N, Jena B (1994). Ageing in fishes. Gerontology.

[CR82] Pauly D (1980). On the interrelationships between natural mortality, growth parameters, and mean environmental temperature in 175 fish stocks. ICES J Mar Sci.

[CR83] Pfannenberg C (2010). Impact of age on the relationships of brown adipose tissue with sex and adiposity in humans. Diabetes.

[CR84] Portner HO (2002). Climate variations and the physiological basis of temperature dependent biogeography: systemic to molecular hierarchy of thermal tolerance in animals. Comp Biochem Physiol A.

[CR85] Ragland SS, Sohal RS (1975). Ambient temperature, physical activity and aging in the housefly, *Musca domestica*. Exp Gerontol.

[CR86] Rikke BA, Johnson TE (2004). Lower body temperature as a potential mechanism of life extension in homeotherms. Exp Gerontol.

[CR87] Rikke BA, Johnson TE (2007). Physiological genetics of dietary restriction: uncoupling the body temperature and body weight responses. Am J Physiol Regul Integr Comp Physiol.

[CR88] Roth GS (2002). Biomarkers of caloric restriction may predict longevity in humans. Science.

[CR89] Sanchez-Alavez M, Alboni S, Conti B (2011). Sex-and age-specific differences in core body temperature of C57Bl/6 mice. Age.

[CR90] Sarup P, Sørensen P, Loeschcke V (2014). The long-term effects of a life-prolonging heat treatment on the *Drosophila melanogaster* transcriptome suggest that heat shock proteins extend lifespan. Exp Gerontol.

[CR91] Soare A, Cangemi R, Omodei D, Holloszy JO, Fontana L (2011). Long-term calorie restriction, but not endurance exercise, lowers core body temperature in humans. Aging (Albany NY).

[CR92] Speakman JR (2005). Body size, energy metabolism and lifespan. J Exp Biol.

[CR93] Speakman JR (2004). Uncoupled and surviving: individual mice with high metabolism have greater mitochondrial uncoupling and live longer. Aging Cell.

[CR94] Spindler SR (2010). Caloric restriction: from soup to nuts. Ageing Res Rev.

[CR95] Sramkova D (2007). The UCP1 gene polymorphism A-3826G in relation to DM2 and body composition in Czech population. Exp Clin Endocrinol Diabetes.

[CR96] Tacutu R (2013). Human ageing genomic resources: integrated databases and tools for the biology and genetics of ageing. Nucleic Acids Res.

[CR97] Trudgill DL, Honek A, Li D, Van Straalen NM (2005). Thermal time-concepts and utility. Ann Appl Biol.

[CR98] Turbill C, Bieber C, Ruf T (2011). Hibernation is associated with increased survival and the evolution of slow life histories among mammals. Proc R Soc B.

[CR99] Valenzano DR, Terzibasi E, Cattaneo A, Domenici L, Cellerino A (2006). Temperature affects longevity and age-related locomotor and cognitive decay in the short-lived fish *Nothobranchius furzeri*. Aging Cell.

[CR100] Van Voorhies WA, Ward S (1999). Genetic and environmental conditions that increase longevity in *Caenorhabditis elegans* decrease metabolic rate. Proc Natl Acad Sci USA.

[CR101] Waalen J, Buxbaum JN (2011). Is older colder or colder older? The association of age with body temperature in 18,630 individuals. J Gerontol Ser A Biol Sci Med Sci.

[CR102] Walford RL (1983). Maximum lifespan.

[CR103] Walford RL, Liu RK (1965). Husbandry, life span, and growth rate of the annual fish, *Cynolebias adloffi* E. Ahl.. Exp Gerontol.

[CR104] Walford RL, Spindler SR (1997). The response to calorie restriction in mammals shows features also common to hibernation: a cross-adaptation hypothesis. J Gerontol Ser A Biol Sci Med Sci.

[CR105] Wang JJ, Tsai JH (2001). Development, survival and reproduction of black citrus aphid, *Toxoptera aurantii* (Hemiptera : Aphididae), as a function of temperature. Bull Entomol Res.

[CR106] Wang X, Chang Q, Wang Y, Su F, Zhang S (2014). Late-onset temperature reduction can retard the aging process in aged fish via a combined action of an anti-oxidant system and the insulin/insulin-like growth factor 1 signaling pathway. Rejuvenation Res.

[CR107] Warn P, Brampton M, Sharp A, Morrissey G, Steel N, Denning D, Priest T (2003). Infrared body temperature measurement of mice as an early predictor of death in experimental fungal infections. Lab Anim.

[CR108] White CR, Seymour RS (2003). Mammalian basal metabolic rate is proportional to body mass 2/3. Proc Natl Acad Sci USA.

[CR109] Wilkinson GS, South JM (2002). Life history, ecology and longevity in bats. Aging Cell.

[CR110] Xiao R, Zhang B, Dong Y, Gong J, Xu T, Liu J, Xu X (2013). A genetic program promotes *C. elegans* longevity at cold temperatures via a thermosensitive TRP channel. Cell.

[CR111] Zhang L, Lu X (2012). Amphibians live longer at higher altitudes but not at higher latitudes. Biol J Linn Soc.

[CR112] Zhou Z-S, Guo J-Y, Chen H-S, Wan F-H (2010). Effects of temperature on survival, development, longevity, and fecundity of *Ophraella communa* (Coleoptera: Chrysomelidae), a potential biological control agent against *Ambrosia artemisiifolia* (Asterales: Asteraceae). Environ Entomol.

